# The MRI features of renal inflammatory pseudotumor: A case report and literature review

**DOI:** 10.1097/MD.0000000000033287

**Published:** 2023-03-24

**Authors:** Yang Han, Guoqiang Yang, Jiangfeng Du, Yan Tan, Hui Zhang

**Affiliations:** a Department of Radiology, First Hospital of Shanxi Medical University, Taiyuan, Shanxi Province, China; b College of Medical Imaging, Shanxi Medical University, Taiyuan, Shanxi Province, China; c Shanxi Key Laboratory of Intelligent Imaging and Nanomedicine, First Hospital of Shanxi Medical University, Taiyuan, Shanxi Province, China.

**Keywords:** CT, immunoglobulin G4-related disease, inflammatory pseudotumor, kidney, MRI, renal inflammatory pseudotumor

## Abstract

Inflammatory pseudotumor is rare observed in renal immunoglobulin G4-related disease.

**Patient concerns:**

A 65-year-old female presented with a mass in the right kidney which was found in physical examination.

**Diagnoses:**

Based on the imaging findings and clinical manifestations, we preliminarily judged that the mass of the right kidney was renal cell carcinoma.

**Interventions:**

The patient finally underwent total nephrectomy.

**Outcomes:**

The final result of microscopic pathological examination is renal inflammatory pseudotumor.

**Lessons:**

There are some characteristics on magnetic resonance imaging of renal inflammatory pseudotumor, which can improve diagnosis rate by combining with medical history and clinical manifestations.

## 1. Introduction

Clinically, the renal inflammatory pseudotumor (RIP) in renal immunoglobulin G4-related disease (IgG4-RD) is very rare.^[1]^ At present, there are many causes of RIP have not yet been elucidated, IgG4-RD may be one of its causes.^[2]^ Correct preoperative diagnosis can avoid total nephrectomy.^[3]^ However, the preoperative imaging findings mostly suggest malignant behavior and urologists often pick up the wrong treatment because of misdiagnosis.^[4]^ From 1999 to 2022, we retrieved 49 English cases of RIP in the Pubmed database. And our case was to summarize characteristics of RIP from 3 perspectives including history and clinical manifestation, imaging findings and histopathology.

Previous cases described 37 cases of computed tomography (CT) and 16 cases of magnetic resonance imaging (MRI) imaging findings. Few literatures summarized the MRI imaging features of RIP. So we hope that we can provide favorable diagnostic clues by digging deeper into MRI characteristics through literature review by combining our case.

## 2. Case report

A 65-year-old female presented with a mass in the right kidney which was found in physical examination. No abnormalities in clinical manifestations. Increased C-reactive protein (53.4 mg/L, normal value < 8 mg/L), gamma globulin (22 g/L, normal value 0.4–3.45 g/L) and accelerated erythrocyte sedimentation rate (33 mm/hour, normal value 0–20 mm/hour) detected in laboratory.

**Figure 1.**
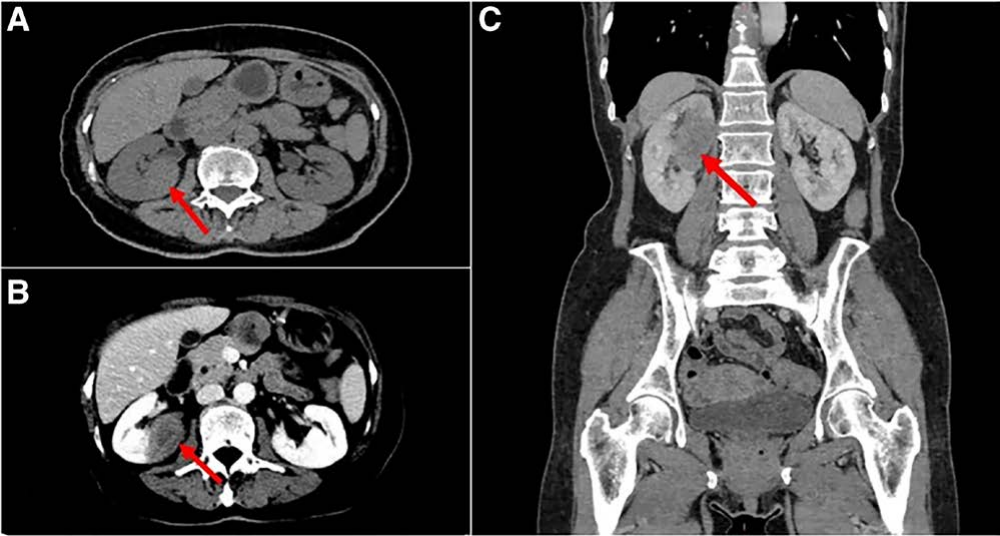
Computerized Tomography (CT) (slice thickness 0.9 mm). (A) Nonenhanced CT shows a homogeneous isodensity mass in the upper portion of right kidney (arrow). (B) Enhanced CT demonstrated mild enhancement in the cortical phase(arrow). (C) In the delayed phase, the enhancement of the mass increases(arrow).

**Figure 2.**
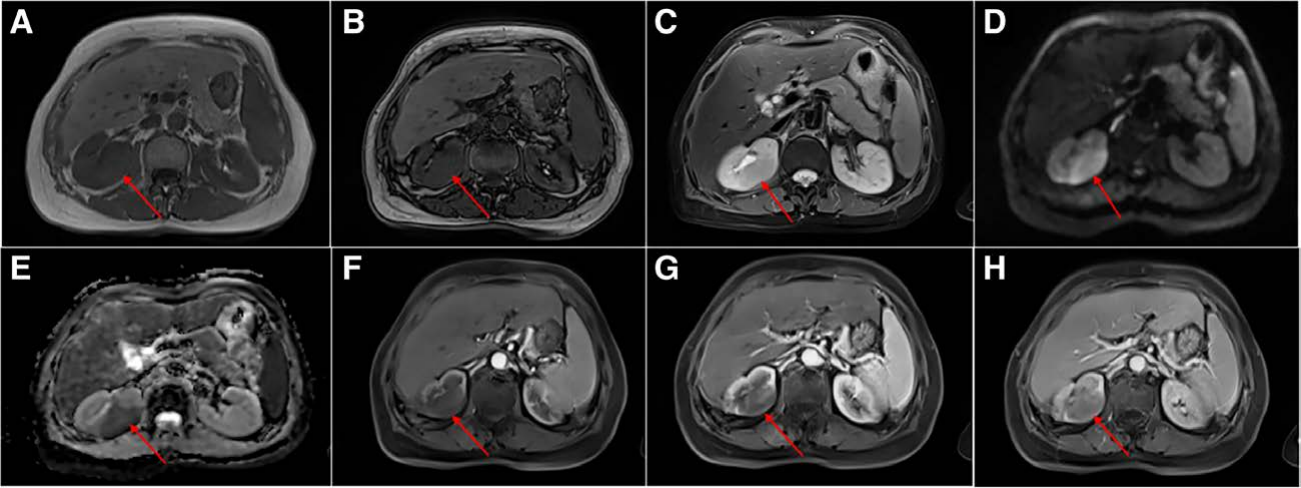
Magnetic resonance imaging (MRI) (slice thickness 6.5 mm). (A) In-phase (arrow). (B) Out-phase (arrow). (C) T2-wighted image shows low signal intensity (arrow). (D) DWI (arrow). (E) ADC (arrow). (F) Arterial phase (arrow). (G) Venous phase (arrow). (H) delay phase (arrow). ADC = apparent diffusion coefficient.

After that, the patient underwent CT and MRI examinations. Plain CT showed a homogeneous isodensity mass in the upper portion of the right kidney with unclear boundary and the average CT value was approximately 40 HU. Enhanced CT demonstrated mild enhancement in the cortical phase, and the CT values of the 3 phases were about 42 HU, 57 HU and 53 HU, respectively (Fig. 1). MRI showed that the mass was slightly low signal on *T*1 and *T*2, and the signal was uniform. The diffusion-weighted imaging (DWI) showed slightly high signal and the apparent diffusion coefficient (ADC) value (b = 800) is about 1.0. The mass was slightly enhanced in the arterial phase and increased in the venous phase and delayed phase (Fig. 2). The size of the mass was 3.7 cm × 2.9 cm × 2.8 cm. The ultimate medical imaging diagnosis was renal cell carcinoma.

**Figure 3.**
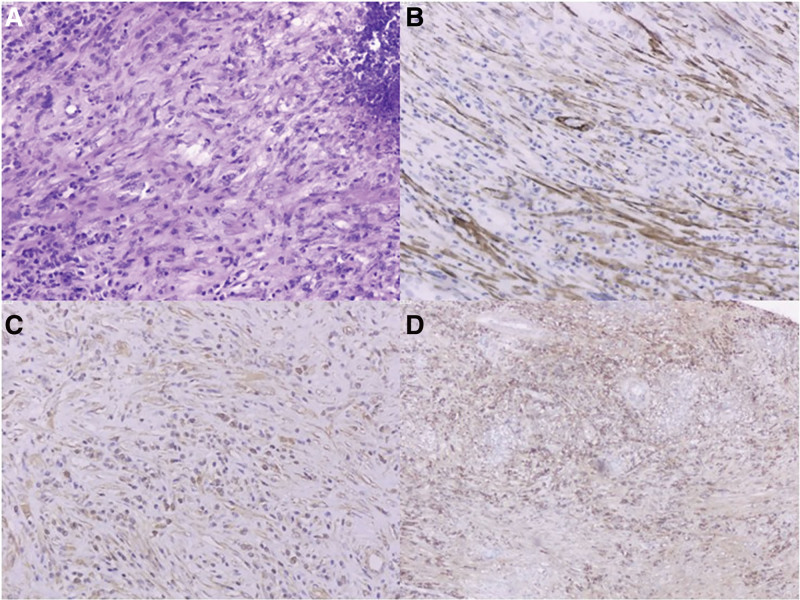
Microscopic findings. (A) A large number of spindle cells as background, and diffuse infiltration of lymphocytes and plasma cells was scattered throughout the lesion (magnification, ×100). (B) The tumor cells were positive for SMA (magnification, ×100) and (C) Vimentin (magnification, ×100). (D) Anti immunoglobulin G4 antibody (magnification, ×40).

The patient underwent radical right nephrectomy under general anesthesia. The pathological result was RIP in renal IgG4-RD. The cell H&E staining sections under the microscope showed that the lesions were backgrounded by a large number of spindle cells, and diffuse infiltration of lymphocytes and plasma cells was scattered throughout the lesion. Immunohistochemical results showed Vimentin, SMA, CD3, CD20, CD21, CD38, CD68, CD138, LCA positive, anaplastic lymphoma kinase1, Desmin negative, Ki67 5%. Pathological diagnosis as inflammatory pseudotumor. (Fig. 3)

## 3. Discussion

We retrieved 49 English case reports of RIP in the PubMed database between 1999 and 2022, all but 2 cases^[5,6]^ were misdiagnosed as malignancy which all except 2 patients who adopted palliative care were treated surgically. Patients could be treated conservatively with drugs, such as steroid hormone therapy, if diagnosed correctly.^[3]^ The high misdiagnosis rate of RIP leaved us with the problem of seeking diagnostic clue before surgery, especially in imaging findings. Now, we would open up discussion from 3 perspectives.

### 3.1. History and clinical manifestations

We conducted a summary of 49 cases about their history and clinical manifestations in Table 1. We found no relationship between age and RIP which was consistent with previously report.^[7]^ There were 2 reports have mentioned that RIP had a high incidence rate among women^[8]^ or man^[9]^ but we thought we should be careful not to draw hasty conclusions because of small sample size. In clinical manifestation, the loin or abdominal pain, fever, wight loss, and hematuria were more commonly seen but the fever could have some diagnostic utility in RIP, which was also noted by Epaulard et al.^[10]^ Nevertheless, there were 11 patients without any symptoms at the same time from Table 1. Boo et al^[11]^ thought the presence of elevated platelet counts and anemia also suggested the RIP but we found a case with reduced platelet counts which was likely to be associated with the primary diseases.^[12]^ We also found that 8 patients had abnormal immune indicators and 19 patients combined with other diseases including IgG4-related disease(IgG4-RLD),^[2,5,13–17]^ Adamantiades-Behçet’s Disease,^[18]^ infection by Histoplasma capsulatum^[19]^ and Epstein-Barr virus,^[12]^ chronic pachymeningitis,^[20]^ primary amenorrhea,^[1]^ chronic Hepatitis B and Raynaud’s disease,^[4,21]^ ipsilateral perinephric and periureteric fibrosis,^[8]^ rheumatoid arthritis,^[22]^ Wegener’s granulomatosis ^[23]^ and gout.^[24]^So it was not difficult to find that immune system abnormalities including infection and autoimmune diseases had a potential relationship with RIP.

**Table 1:** Epidemiological and pathological results of the 49 cases in the literature review.

Item	Statistical result
Age range	9 mo–80 yr
Sex (F:M)	26:22
Location (RK:LK)	28:21
Symptoms	
Loin/abdominal pain	24
Fever	10
Wight loss	8
Hematuria	10
Asymptomatic	11
Night sweats	2
Laboratory findings	
Anemia	8
Platelet count (E:R)	4:1
Abnormal renal function	5
Abnormal immune indicators	8
Combined with other diseases	19

E = elevated, F = female, LK = left kidney, M = male, R = reduce, RK = right kidney.

To sum up, we have found that patients with RIP may have inflammation-related clinical manifestations or experimental abnormalities and abnormal immune indicators.

### 3.2. Histopathology

The initial cause of the RIP is likely to be the renal immune complex deposition in most cases.^[21]^Otherwise, there are certainly some reasons for rearrangements involving anaplastic lymphoma kinase gene that the literature appears to support.^[21,25]^ Grossly, the presentation of mass, especially the formation of vessels, depends on weak or strong inflammatory response.^[2]^ Inflammation lasting for a long time can induce angiogenesis which is vulnerable and easy to bleed.^[26]^ Under the microscope, the histologic examination of most cases reveals the bulk of the mass to contain spindle cells admixed with collagen, lymphocytes and plasma cells which it is also known as inflammatory myofibroblastic tumor.^[25]^ The spindle cells which have myofibroblastic features and are of fibroblastic or myofibroblastic origin express Vimentin and SMA.^[3]^ However, the histologic examination of few cases revealed lymphoplasmacytic infiltration without spindle cells.^[16]^ Either way, the mass is the inflammatory, fibrosis will be the end of chronic inflammatory reactions.^[2]^

In any case, the mass always has the characteristics of fibrosis, which is also the pathological basis for determining the characteristics of imaging findings.

### 3.3. Imaging findings

Among these 49 cases, 37 received routine CT examinations. On CT scans, RIP presented low density, isodensity or high density due to the weak or strong inflammatory response.^[27]^Even though the mass could be either solid, cystic or solid–cystic in nature, the most case all mentioned the solid tissue of mass presented mild enhancement during the arterial for existence of fibrous components.^[28]^There was 16 cases received routine MRI examinations and we conducted a summary in Table 2. First, these cases all mentioned the mass showed low signal intensity on *T*2 for which the mass have spindle cells. Second, RIP all presented delayed enhancement which was probably due to an accumulation of extravascular contrast media in the fibrotic component of the lesion.^[29]^ Finally, there were 2 cases mentioned that diffusion-weighted imaging (DWI, b value = 800) of IPT showed slightly hyperintensity.^[2,16]^ Our patient’s case was mostly same as mentioned above. So we thought RIP should be suspected when the above 3 characteristics appeared in MRI, especially the DWI and ADC.^[30]^ Diffusion MRI studies yielded significantly different ADC between benign and malignant renal masses but few case reports described it.^[30]^ The ADC value (b = 800) of our case was about 1.0. Thus, we appeal to radiologists to report more MRI findings of RIP so that we can find features and make accurate diagnosis.

**Table 2:** Reported renal RIP (only cases in which MRI were performed).

Case	Year	Age	Sex	Location	Number	Boundary	Nature	T1WI	T2WI	Enhancement	DWI
1	1999	56 yr	F	LK	Solitary	Ambiguous	Solid	L	L	NM	NM
2	2004	38 yr	M	LK	Solitary	Ambiguous	Solid	L	L	NM	NM
3	2005	46 yr	M	RK	Solitary	Ambiguous	Solid	L	NM	NM	NM
4	2007	61 yr	M	LK	Solitary	Ambiguous	Solid	ISO	L	DE	NM
5	2009	24 yr	F	LK	Solitary	Ambiguous	Cystic	NM	NM	DE	NM
6	2010	59 yr	M	RK	Solitary	Ambiguous	Cystic compactness	NM	L	DE	NM
7	2010	61 yr	F	LK	Solitary	Ambiguous	Solid	NM	L	NM	NM
8	2010	56 yr	F	LK	Solitary	Ambiguous	Solid	L	L	NM	NM
9	2011	48 yr	M	RK	Solitary	Ambiguous	Cystic compactness	NM	H	DE	NM
10	2013	18 yr	F	LK	Solitary	Ambiguous	Solid	L	L	NM	NM
11	2018	3 yr	M	RK	Solitary	Ambiguous	Solid	NM	L	DE	NM
12	2019	69 yr	F	RK	Solitary	Ambiguous	Solid	L	L	DE	Slightly high
13	2020	35 yr	F	RK	Solitary	Ambiguous	Solid	L	L	DE	NM
14	2021	13 yr	F	RK	Solitary	Ambiguous	Solid	L	H	DE	NM
15	2022	9 mo	F	RK	Solitary	Ambiguous	Solid	NM	NM	DE	NM
16	2022	60 yr	M	LK	Solitary	Ambiguous	Cystic compactness	L	L	DE	Slightly high

DE = delay enhancement, DWI = diffusion-weighted imaging, F = female, H = high signal intensity, ISO = iso-signal intensity, L = low signal intensity, LK = left kidney, M = male, MRI = magnetic resonance imaging, NM = not mentioned, RK = right kidney, RIP = renal inflammatory pseudotumor.

The differential diagnosis of RIP may include malignant tumors such as papillary renal cell carcinoma, transitional cell carcinoma, inflammatory fibrosarcoma, malignant fibrous histiocytoma and nonmalignant tumors such as angiomyolipoma, xanthogranuloma pyelonephritis and plasma cell granuloma because of low signal on *T*2.

## 4. Conclusion

We find many common characteristics in literature review and summary about RIP. First of all, the immune system of more patients is abnormal or they suffer from immune-related diseases and the relevant inflammatory indicators and immunological indicators are also abnormal. Second, the presence of spindle cells is the most important feature of RIP, which is the basis of imaging findings. Finally, there are some characteristics on MRI of RIP, which can improve diagnosis rate by combining with medical history and clinical manifestations.

## Author contributions

**Conceptualization:** Guoqiang Yang, Jiangfeng Du, Yan Tan.

**Funding acquisition:** Hui Zhang.

**Investigation:** Yang Han.

**Methodology:** Yang Han.

**Resources:** Guoqiang Yang.

**Software:** Jiangfeng Du.

**Supervision:** Jiangfeng Du.

**Validation:** Yang Han.

**Visualization:** Yang Han.

**Writing – original draft:** Yang Han.

**Writing – review & editing:** Yan Tan, Hui Zhang.
